# Assessing Methods for Evaluating the Number of Components in Non-Negative Matrix Factorization

**DOI:** 10.3390/math9222840

**Published:** 2021-11-09

**Authors:** José M. Maisog, Andrew T. DeMarco, Karthik Devarajan, S. Stanley Young, Paul Fogel, George Luta

**Affiliations:** 1 Blue Health Intelligence; 2 Department of Rehabilitation Medicine, Georgetown University Medical Center; 3 Department of Biostatistics and Bioinformatics, Fox Chase Cancer Center, Temple University Health System, Philadelphia, PA 19111; 4 GCStat, 3401 Caldwell Drive, Raleigh, NC 27607; 5 Advestis, 69 Boulevard Haussmann 75008 Paris, France; 6 Department of Biostatistics, Bioinformatics and Biomathematics, Georgetown University Medical Center; 7 Department of Clinical Epidemiology, Aarhus University, Aarhus, Denmark; 8 The Parker Institute, Copenhagen University Hospital, Frederiksberg, Denmark

**Keywords:** Non-negative matrix factorization, normalization, PCA, factorization rank, number of factored components, high-dimensional data, unsupervised learning

## Abstract

Non-negative matrix factorization is a relatively new method of matrix decomposition which factors an m×n data matrix X into an m×k matrix W and a k×n matrix H, so that X≈W×H. Importantly, all values in X, W, and H are constrained to be non-negative. NMF can be used for dimensionality reduction, since the k columns of W can be considered components into which X has been decomposed. The question arises: how does one choose k? In this paper, we first assess methods for estimating k in the context of NMF in synthetic data. Second, we examine the effect of normalization on this estimate’s accuracy in empirical data. In synthetic data with orthogonal underlying components, methods based on PCA and Brunet’s Cophenetic Correlation Coefficient achieved the highest accuracy. When evaluated on a well-known real dataset, normalization had an unpredictable effect on the estimate. For any given normalization method, the methods for estimating k gave widely varying results. We conclude that when estimating k, it is best not to apply normalization. If underlying components are known to be orthogonal, then Velicer’s MAP or Minka’s Laplace-PCA method might be best. However, when orthogonality of the underlying components is unknown, none of the methods seemed preferable.

## 1. Introduction

Matrix decomposition methods [1]–[3] are an important area of study in mathematics, and encompasses approaches to factoring an observed matrix into a mixture of other matrices. This addresses a common challenge in environmental and public health research where data is measured empirically as a mixture of source signals, but it is important to unmix the data to understand the underlying structure of the phenomenon being studied.

NMF is an unsupervised learning approach used to perform matrix decomposition, and requires that the number of unmixed components be supplied by the experimenter. Yet, the number of underlying components is often unknown and, indeed, it is not clear the optimal approach to determining the correct number. Moreover, data is typically preprocessed, including normalization, prior to applying the NMF procedure. Similarly, it is not clear what normalization procedure is optimal. Here we formally evaluate existing rank selection methods based on various normalization schemes in the context of NMF. We are not aware of a paper that specifically addresses the issue of rank selection and data normalization within the context of NMF, and believe this is the first of its kind dealing with this important problem.

The NMF approach has found a range of uses in both environmental science and public health, with various computational implementations. For instance, Jiang et al. [4] employed a concordance method to discover five stable factors shaping the family structure of ocean microbes based on genomic sequencing. Sutherland-Stacey and Dexter [5], used projected gradient descent to identify two chemical factors corresponding to pollutants in the spectra measured from dairy processing wastewater. Ramanathan et al. [6] used the alternate least squares algorithm to identify and characterize five geo-temporal patterns explaining the co-occurrence of asthma and flu based on ZIP code. In the context of public health, Stein-O’Brien et al. [7] demonstrates the application of NMF to gene-expression data and reviews its utility in addressing questions ranging from systems-level to cell-level analysis in genetics. Liu et al. [8] demonstrated a graph-regularized implementation to identify 38 factors linking microbes and their associated diseases. Applications of NMF are not limited to –omics data, as evidenced by a recent effort in which Luo et al. [9] used alternating least squares with projected gradient descent to capture 13 latent topics related to suicidality in social media.

Some decomposition methods such as the Cholesky decomposition, the Lower-Upper decomposition, the QR decomposition, and Singular Value Decomposition (SVD) provide a means for computing the inverse or pseudoinverse (generalized inverse) of a square matrix, or for solving a system of simultaneous linear equations (e.g., see Chapter 2 of [10]). Other decomposition methods provide a way to cluster or summarize data, that is, to reduce dimensionality. A classic example is Principal Components Analysis (PCA), which is closely related to SVD, and which constrains its components to be orthogonal (for applications, see [11], e.g.). Another example is Independent Components Analysis (ICA) (for applications, see [12], [13], e.g.), which instead constrains its components to be statistically independent. *Non-negative matrix factorization* (NMF) [14]–[16] is a relatively new matrix decomposition method. NMF factors an *m*×*n* non-negative data matrix *X* into an *m*×*k* matrix *W* and a *k*×*n* matrix *H* such that

(1) X=W×H+e≈W×H

where *e* is an *m*×*n* matrix of approximation errors, and where *k* is chosen such that (*n*+*m*)*k*<*nm*, i.e., *k* is less than both *m* and *n* [15], [16]. Importantly, NMF constrains all three matrices, *X*,*W*, and *H*, to have only non-negative elements; hence the term *non-negative* matrix factorization.

Much like PCA, NMF can be used to reduce dimensionality. However, unlike PCA, the NMF approach can account for a hierarchical structure [17]. NMF has an advantage over standard hierarchical clustering (HC; for an introduction, see [18]): whereas HC forcibly imposes a hierarchical structure on data, even when no such hierarchical structure is present, NMF will refrain from such Procrustean behavior.

In their seminal paper introducing the NMF approach, Lee and Seung [16] provided the following recurrence relation to estimate a solution to Equation 1:

(2) $$W_{ia}\leftarrow W_{ia}\sum_{j}\frac{X_{ij}}{{\left(W\;H\right)}_{ij}}H_{aj}$$

(3) $$W_{ia}\leftarrow \frac{W_{ia}}{\sum_{j}W_{ja}}$$

(4) $$H_{aj}\leftarrow H_{aj}\sum_{i}\frac{X_{ij}}{{\left(W\;H\right)}_{ij}}W_{ia}$$

Matrices *W* and *H* are usually initialized with non-negative pseudorandom values; but note that some researchers have examined the effect of initializing with more carefully selected values [19]–[22]. A possible stopping criterion for Equations 2–4 might be defined as follows. The *Kullback-Liebler* criterion [15], [16] is:

(5) $$KL(X|\left|WH\right)=\sum_{ij}\;[X_{ij}\;log\frac{X_{ij}}{{\left(W\;H\right)}_{ij}}-X_{ij}+{\left(WH\right)}_{ij}]$$

Brunet’s MATLAB implementation of NMF minimizes this criterion [23]).

Define

(6) $$\delta_{\kappa +1}=D_{\kappa +1}-D_{\kappa }$$

where *D_κ_* is the Kullback-Liebler criterion (Equation 5) evaluated at the *κ^th^* iteration. Iterate Equations 2–4 until *δ_κ_* (Equation 6) falls below some threshold value. An alternative criterion to minimize is the *squared Euclidean distance*

(7) $$E2(X\|\;WH)=\sum_{ij}{\left[X_{ij}-{\left(WH\right)}_{ij}\right]}^{2}=||X-WH|{|}_{F}^{2}$$

where ${\left||\cdot |\right|}_{F}$ is the Frobenius norm [24] (or alternatively called the Euclidean norm).

While the choice of criterion *is* relevant to the distribution of the data at hand, Lee and Seung state that this choice is not as important as the non-negativity constraints “for the success of NMF in learning parts” [16], and that the use of the Kullback-Liebler criterion may have computational advantages over the squared Euclidean distance, especially for larger data sets [16].

Since Lee and Seung’s paper, newer methods for computing the NMF have been devised. Lin’s *projected gradients method* is based on a Euclidean metric [24]. Kim and Park suggest a fast approach based upon a *block principal pivoting method* [25], [26].

Of note, the computed non-negative factorization is not unique. Different answers could be obtained depending on the initialization of the matrices *W* and *H*. Moreover, each different initialization may obtain distinct local minima in the search space of the criterion.

In this manuscript, we will use *k*_0_ to denote the *true* underlying number of components. On the other hand, $\hat{k}$ will represent an *estimation* of *k*_0_ by one of the methods tested. Evaluating $\hat{k}$ will mean the same thing as estimating *k*_0_.*k* will simply mean a possible number of underlying components.

With NMF, one must choose the number of components *k* into which one wants to decompose a matrix *X* (Equation 1). This requirement is analogous to the situation in *k*-means clustering, in which one must choose *a priori* the number of desired clusters *k*. Indeed, the link between k-means clustering and NMF goes beyond the superficial similarity of needing to choose *a priori* the number of desired components or clusters. A deeper link has been shown between the algorithms. Specifically, Ding, He, & Simon (2005) [27] initially claimed that symmetric NMF was equivalent to kernel k-means. Later, Kim & Park (2008) [28] showed that by placing certain constraints on the NMF formulation, it becomes equivalent to a variation of k-means clustering.

Because the number of true underlying components k is often unknown in practice, and given that NMF is an unsupervised learning method, the importance of estimating k accurately is self-evident. The proper value for k depends on the natural underlying properties of the phenomenon under investigation, but as noted above this is often unknown. If k is chosen to be too small, then potentially-important clustered structures in the data are missed, and the original goal of NMF to reduce the dimensionality of the dataset in a meaningful way is not achieved. If k is too large, then these important components may become excessively fragmented and difficult to study or interpret. Yet, despite the importance of choosing an appropriate k, it is unclear what is the best way for estimating k, and moreover we are not aware of a paper that specifically addresses the issue of rank selection and data normalization within the context of NMF.

The need to choose k *a priori* can be argued to be similar to the situation in PCA and ICA, in which one must decide *a posteriori* which components to consider true signal, and which to consider as merely noise. With PCA, one might use Cattell’s scree test [29] or Kaiser’s rule [30], or perhaps newer methods such as *Velicer’s Minimum Average Partial (MAP)* method [31], [32] and *Minka’s Laplace-PCA* and *BIC-PCA methods* [33]. Similar methods have been developed in the ICA context as well; see, for example, Li et al., 2007 [34].

Three methods for evaluating *k* that were developed in the context of PCA are:

- **Velicer’s Minimum Average Partial (MAP) method** [31], [32]. In this method, a complete PCA is performed. Then, a stepwise assessment of a series of *N* − 1 matrices of partial correlations is performed, where *n* is the number of principal components. In the *p^th^* such matrix, the first *p* principal components are partialed out of the correlations. Then, the average squared coefficient in the off-diagonals of the resulting partial correlation matrix is computed. Components are retained if the variance in the matrix of partial correlations is judged to represent systematic variance. For a full description of this method, see the original papers [31], [32] as well as O’Connor [35]).
- **Minka’s Laplace-PCA method** [33]. In this method, Minka uses Bayesian model selection to estimate *k*_0_. He uses Laplace’s method [36] to approximate an integral which would otherwise be difficult to compute analytically. See Equation 80 of Minka’s technical report for details.
- **Minka’s BIC-PCA method** [33]. This is a variant of Minka’s Laplace-PCA method in which a second approximation is made that further simplifies the computation. See Equation 82 of Minka’s technical report for details.

The following four methods are based on criteria that must be numerically optimized, and are thus considered “iterative” methods in this paper. Note that they contain a squared difference term; they are thus based on the Frobenius norm. For Poisson-distributed data, or for use with NMF computed using the Kullback-Liebler criterion (Equation 5), the squared difference term should be replaced with the Kullback-Liebler criterion. For normally distributed data, it might be best to retain the squared difference term.

- **Three Bayesian Information Criterion (BIC) methods.** Let *W*^(*k*)^ and *H*^(*k*)^ be the result of computing the NMF as per Equation 1, where *k* is some possible number of underlying components, i.e., *W* is *m*×*k* and *H* is *k*×*n*. Further, let ${\hat{X}}^{\left(k\right)}=W^{\left(k\right)}\times H^{\left(k\right)}$. Then three model selection criteria similar to the Bayesian Information Criterion ([37]; see [38] for review) are [39], [40]:
(8) $$BIC1\left(k\right)=\text{log}({{||\hat{X}}^{\left(k\right)}-X||}^{2})\;+\;k\frac{m+n}{mn}\text{log}(\frac{mn}{m+n}),$$
(9) $$BIC2\left(k\right)=\text{log}({{||\hat{X}}^{\left(k\right)}-X||}^{2})\;+k\frac{m+n}{mn}\text{log}(c^{2}),$$
(10) $$BIC3\left(k\right)=\text{log}({{||\hat{X}}^{\left(k\right)}-X||}^{2})\;+k\frac{m+n}{mn}\frac{\log\left(c^{2}\right)}{c^{2}},$$
  Where $c=c\left(m,n\right)=\min\left(\sqrt{m},\sqrt{n}\right),\;$ and $||A||={\left[tr(A^{'}A)\right]}^{1/2}={\left||A|\right|}_{F}\;$[39], [40].
- **Shao’s relative root of sum of square differences (RRSSQ).** With ${\hat{X}}^{\left(k\right)}$ as defined above, Shao et al. suggest the following optimization criterion [41]:
(11) $$RRSSQ\left(k\right)=\sqrt{\frac{{\left||X-{\hat{X}}_{ij}^{\left(k\right)}|\right|}_{F}^{2}}{\sum_{i}^{m}\sum_{j}^{n}{\left(X_{ij}\right)}^{2}}}$$

Three methods for evaluating the number of underlying components *k* that have been developed in the context of NMF are:

- **Fogel and Young’s volume-based method (FYV)**. Let ${\hat{X}}_{k}$ be ${\hat{X}}^{\left(k\right)}$ reshaped into a column vector, with ${\hat{X}}^{\left(k\right)}$ defined as above. The $\hat{k}$ vectors ${\hat{X}}_{k},\;1\leq k\leq \hat{k}$ are computed, and are each normalized. A $\hat{k}$-column matrix is then constructed from the $\hat{k}$ vectors ${\hat{X}}_{k}$, and the determinant of this matrix is used as the optimization criterion. An abrupt decrease in the value of this determinant (plotted as a function of $\hat{k}$) indicates the best estimate of the underlying components *k*; Fogel and Young use the algorithm of Zhu and Ghodsi [42], originally developed to automate Cattell’s scree test [29], to detect this abrupt decrease. This volume-based method is based on the geometric interpretation of the determinant of an N×N matrix as the volume of a *n*-dimensional parallelepiped ([43], p. 154).
- **Brunet’s cophenetic correlation coefficient method (CCC)** [23]. This method uses the cophenetic correlation coefficient $\rho_{\hat{k}}(\overset{-}{C})\;$ to measure dispersion for the calculated consensus matrix $\overset{-}{C}$, computed specifically as the Pearson correlation between two matrices measuring distance:
  1. *I* − *C*, the distance between samples measured by the distance matrix; and
  2. the distance between samples measured by the linkage used to reorder $\overset{-}{C}$.
    The value of $\hat{k}$ where $\rho_{\hat{k}}(\overset{-}{C})\;$ begins to decrease is selected as the best estimate of *k*.
- **Owen and Perry’s bi-cross-validation method (BCV)** [40]. This method is based on the Frobenius norm criterion given in Equation 7 (see step 8 in the algorithm on page 11 of Owen and Perry’s technical report), uses a truncated SVD, and performs cross-validation across both columns and rows (hence *bi-*cross-validation).

While NMF seems to be a robust algorithm [44], some sort of normalization of the data matrix *X* is usually necessary as a pre-processing step to make the estimated components “more evident” [45]. For that reason, Pascual-Montano et al. have implemented several normalization methods in their bioNMF system [45]. Interestingly, Okun and Priisalu showed that normalization can sometimes reduce the time required to compute the NMF [21] when using Lee and Seung’s original recurrence relation (Equations 2–4) [16]), and with *W* and *H* initialized with non-negative random values. This raises the question whether normalization might affect the estimate of the number of underlying components *k*.

However, although there are instances of normalization by column [4], there is frequently no mention of normalization [5], [6], [8], [9]. An examination of the effect of normalization on the estimation of $k_{0}$ is warranted. Note that after normalization the data may have negative values, so some method for enforcing non-negativity may be necessary. Pascual-Montano et al. suggest four such methods [45]: subtracting the absolute minimum, fold data by rows, fold data by columns, and exponential scaling.

In summary, this paper had two objectives. The first objective was to assess several methods for estimating the number of underlying components *k*_0_ in the context of NMF. The second objective was to examine the effect of various normalization methods on the estimation of *k*_0_. To address the first objective, ten methods for evaluating $\hat{k}$ were assessed on simulated data with a known number of components *k*_0_. To address the second objective, eight normalization methods [45] were applied to a well-known data set [46], and the number of underlying components was then estimated using ten methods. Lin’s method [24] was used to compute the NMF [47].

## 2. Materials and Methods

Several of the methods for estimating *k*_0_ (e.g., Velicer’s MAP [31] and Minka’s Laplace-PCA method [33]) as well as two of the implementations for computing the NMF Lin’s method [24] and Brunet’s method [44]) were already available as MATLAB scripts. For this reason, MATLAB was selected as the language to use for this work. As much as possible, the original MATLAB scripts were used; if modifications were necessary, these were kept to a very minimal level. Fogel and Young’s volume-based method was provided as a JMP code snippet, which was translated to MATLAB. The translation was checked by the original author (P. Fogel) and confirmed to be correct.

We simulated data with a known number of underlying components *k*_0_, and then observed the accuracy of ten different methods for estimating the number of components. To simulate data, we implemented a hybrid of the approaches of Kim and Park [26] and Cichocki et al. [48]. Kim and Park’s basic method was used because it was straightforward and explicitly described, while Cichocki’s idea of using orthogonal components was used to enable recovered components to be easily visualized. Specifically, for *k*_0_ = 2 through 20, we constructed a 100×*k*_0_ matrix *W* with orthogonal columns, and a *k*_0_×1000 matrix *H* containing pseudorandom values generated from a uniform distribution, with 40% sparsity. The 100×1000 matrix *X* = *W* × *H* was then computed, and Gaussian noise with mean zero and *SD* =5% of the average magnitude of elements in *X* was added. All negative values were then forced to be positive by taking the absolute value. The MATLAB implementation of this procedure is included as an appendix A (Generation of Synthetic Data).

Then, the following nine methods for estimating the number of underlying components *k*_0_ were applied to the synthetic data:

- Velicer’s MAP [31].
- Minka’s Laplace-PCA method [33].
- BIC1 [39], [40]
- BIC2 [39], [40]
- BIC3 [39], [40]
- Shao’s relative root of sum of square differences (RRSSQ) [41].
- Fogel and Young’s volume-based method (FYV) [44].
- Brunet’s cophenetic correlation coefficient method (CCC) [44].
- Perry’s BCV Method [40]

1000 datasets were simulated at each true *k*_0_ value (2 to 20). Each method for estimating *k*_0_ was then applied once to each of the 1000 simulated datasets at each true *k*_0_. Minimum reconstruction error was not used to select the run for estimating *W* and *H*. The CCC method was run only once with number of runs specified at 20. Reproducibility across simulations was evaluated using the concordance correlation coefficient [49].

Brunet’s CCC-based approach requires a threshold to be applied to choose the answer from the multiple possibilities tested. We automated this selection procedure as:

1. Compute the CCC for a range of values for $\hat{k}$. Let the CCC value corresponding to a value $\hat{k}$ be $\;CCC_{\hat{k}}$.
2. Find the maximum value of $CCC_{\hat{k}}$ across the range of values for $\hat{k}$; call this *C_max_*.
3. Compute the CCC threshold, *C_thr_* = *C_max_* ⋅ *q*, where *q* is a tuning parameter, 0 < *q* < 1. For example, if you want to allow for peaks that are at least 99.9% of the maximum value *C_max_*, set*q* = 0.999.
4. Find the largest index $\hat{k}$ such that $CCC_{\hat{k}}\;\geq C_{thr}$.

An empirically chosen value of *q*=0.999 was used.

Brunet’s implementation of the CCC requires the user to input the desired number of re-initializations; in the datasets they examined, Brunet et al. found 20–100 re-initializations to be sufficient [44]. In this study, the CCC was re-initialized 20 times. Lin’s method [24] was used to compute the NMF.

The well-known data set of Golub et al. [46] was used to examine the effects of normalization on the estimate of *k*_0_. Briefly, this dataset consists of 72 individuals with cancer with ~7500 genes typed. Only the 5,000 genes with the greatest coefficient of variation were used. The 72 observations were rows, while the 5,000 variables were columns. In the absence of other publicly available datasets, we focus on this dataset because it has been used as a benchmark by many researchers. Although we are interested in applications in environmental science and public health research, we believe it has enough complexity to be practically useful and still relevant to the types of datasets studied in public health and environmental science. Critically, there is agreement on the true underlying number of clusters in this dataset.

The following eight methods were assessed for their effect on estimating *k*:

1. No normalization
2. Scale columns, then normalize rows. See [45], [50] for this method.)
3. Set mean = 0, standard deviation = 1 by rows.
4. Set mean = 0, standard deviation = 1 by columns.
5. Set mean = 0, standard deviation = 1 globally. (This method was not listed by Pascual-Montano et al. [45], but was included for completeness.)
6. Subtract the mean by the rows.
7. Subtract the mean by the columns.
8. Subtract the mean by the rows and then by the columns.

The eight methods listed above were then applied to the data. After certain methods of normalization are applied (e.g., subtracting the mean by rows), some values may be negative. To enforce non-negativity, the global minimum value was subtracted from all matrix entries. Then, the ten methods for estimating *k* listed above were each applied to the normalized data. Lin’s method [24] was again used to compute the NMF.

## 3. Results

### 3.1. Methods for estimating $k_{0}$

#### 3.1.1. Methods based on PCA

**Figure 1.**
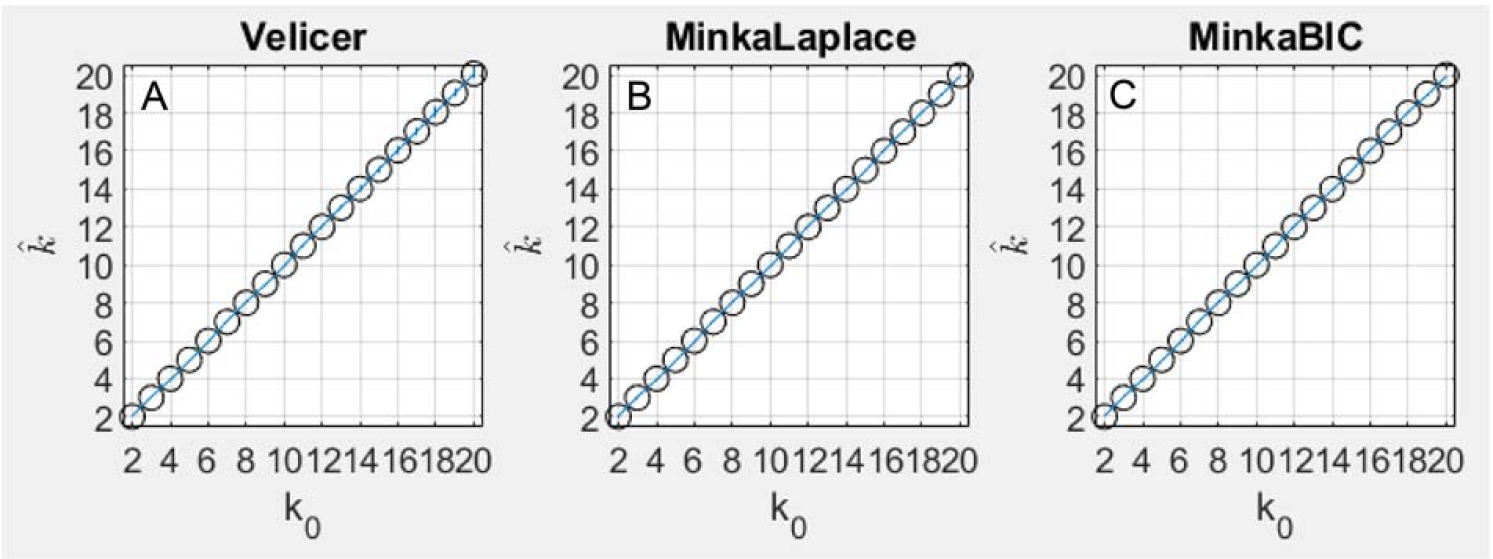
This figure plots the average accuracy result for the three methods based on PCA, including Velicer’s method (A), Minka Laplace method (B), and Minka’s BIC method (C). The results are plotted as the true number of components simulated on each *x* axis and the number of components discovered by each algorithm on each *y* axis. Perfect accuracy should appear as a diagonal line, and indeed that is nearly what each of these three methods achieved. Note that the standard deviation is shown for each estimate by blue error bars, although these errors are small.

With the simulated data, the three methods tested based on PCA (Velicer’s MAP, Minka Laplace, and Minka BIC) closely tracked the number of underlying components in terms of accuracy (Figure 1, A). Velicer’s MAP method was accurate in 100% of simulations up until *k*_0_ = 10, but at *k*_0_ = 10, Velicer’s method began to overestimate *k*_0_ by 1 in a small proportion of stimulations, and this proportion grew to 7% of simulations at *k*_0_ = 20. Minka’s Laplace method overestimated *k*_0_ by 1 in approximately 0.2% of simulations where *k*_0_ ≤ 3, and was accurate in 100% of simulations where*k*_0_ > 3 (Figure 1, B). Minka BIC was 100% accurate in all simulations for all values of *k*_0_ tested in this paper (Figure 1, C).

#### 3.1.2. Iterative methods

**Figure 2.**
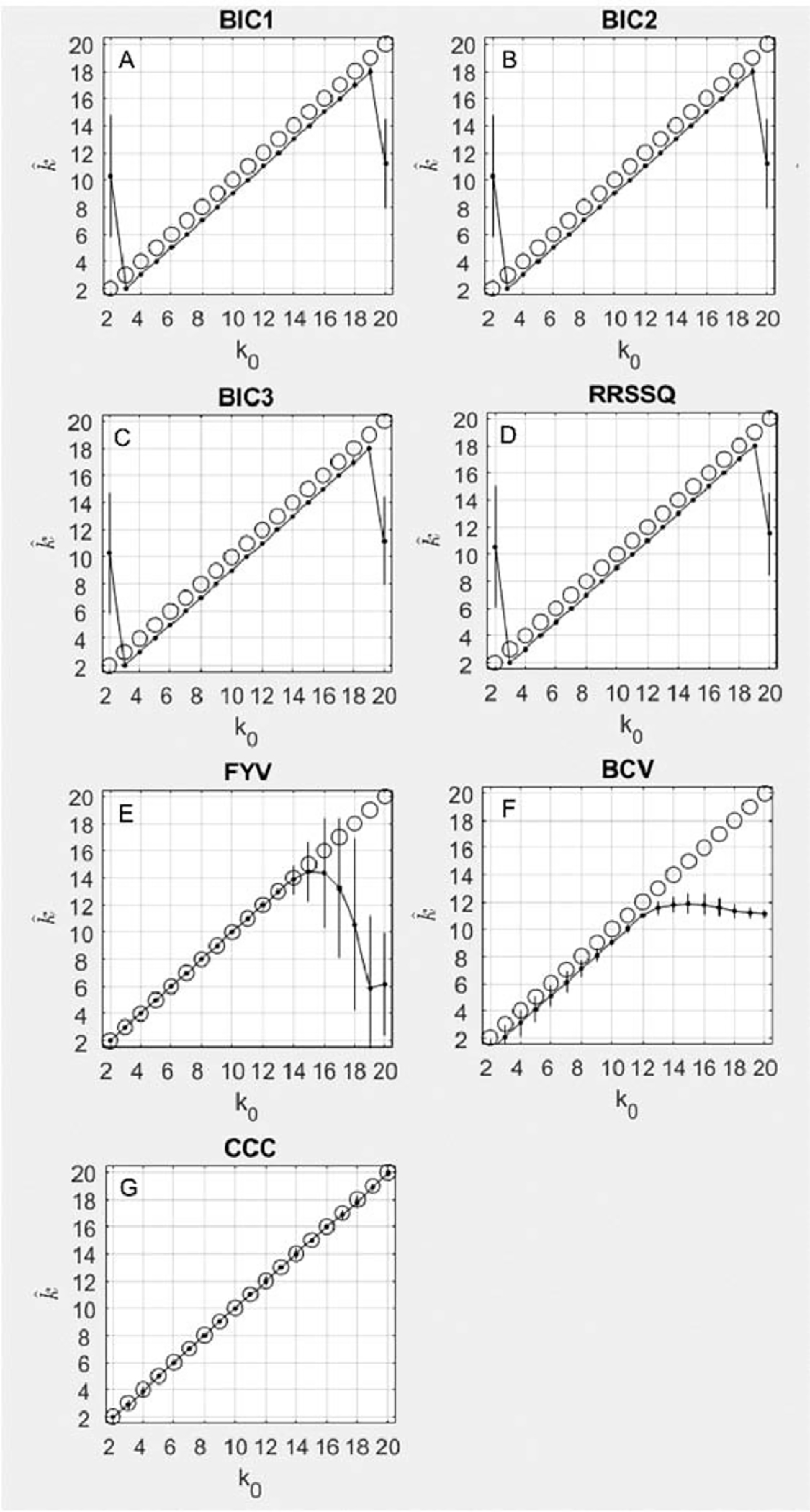
This figure plots the average results for seven iterative methods. The results are plotted as the true number of components simulated on each *x* axis and the number of components discovered by each algorithm on the *y* axis. Perfect accuracy should appear as a diagonal line, following the black circles. The mean result at each *k*_0_ is shown as a black dot and its standard deviation is shown as a black vertical line.

As shown in Figure 2 (A–C), the BIC1, BIC2, and BIC3 results were accurate for all simulations, overestimating by only 1, for all *k*_0_ between 3 and 19. For *k*_0_ = 3 and *k*_0_ = 19, there were no peaks in the response criterion, and consequently the selected $\hat{k}$ was incorrect. The same pattern of accuracy and offset was observed for the RRSSQ method (Figure 2D). The three BIC methods achieved numerically identical concordance correlation coefficients of .98 (95% CI = 0.94 – 0.99), while the RRSSQ method achieved a similar concordance correlation coefficient of .98 (95% CI = 0.94 – 0.99)

#### 3.1.3 NMF Methods

For the FYV method (Figure 2E), the best estimate of the number of underlying components *k*_0_ is found by an abrupt decrease in the value of the determinant plotted as a function of $\hat{k}$. The inflection point was chosen as the first element followed by a decrease with a magnitude greater than 25% of the average decrease for the $\hat{k}$ vector. The FYV method achieved 100% accuracy for all simulations where *k*_0_ < 15. For *k*_0_ = 15, the method began to estimate a progressively lower $\hat{k}$ with a greater degree of variability (note error bars). The FYV method achieved a concordance correlation coefficient of .88 (95% CI = 0.71 – 0.95). The BCV method’s accuracy (Figure 2F) was on average one index short of the simulated *k*_0_, but remained accurate up to around *k*_0_ = 12, at which point the estimate plateaued around 12 for the remainder of values of *k*_0_. The BCV method achieved a concordance correlation coefficient of .99 (95% CI = 0.98 – 0.997). The CCC method’s accuracy (Figure 2G) was on average perfect across the full range of simulated $k_{0}$, achieving a concordance correlation coefficient of .998 (95% CI = 0.995 – 0.999).

### 3.2. Effects of normalization

For each method of normalization, the result of estimating *k*_0_ using various methods is shown in Table 1 below. For any given method for estimating *k*_0_, the choice of normalization method appears to have an unpredictable effect on the estimate. In addition, for any given normalization method, the methods for estimating *k*_0_ in general give widely varying results. It should be noted that the FYV method is the only approach which consistently returns 4 components, which is thought to be a biologically sound number for this particular data set [23], [44].

## 4. Discussion

Matrix decomposition methods allow mixtures of signals to be separated into their original components, but it is often unclear how many components to choose. We explored this question by simulating signal mixtures and testing various matrix decomposition methods on them to estimate the number of underlying components. We also explored the effect of normalization on estimates of the number of components.

We found that the three methods based on PCA that we tested consistently and accurately measure the true number of simulated components. The four iterative methods tested also performed well, but estimates at the boundaries of their “guessing range” were inaccurate. In contrast, the NMF-based methods differed in their accuracy. While the CCC method achieved perfect accuracy across all simulated values of k, the FYV and BCV method became inaccurate around k = 15 and 13, respectively. This likely relates to the heuristic for choosing the inflection point for $\hat{k}$. One possibility is that point at which these two methods start becoming inaccurate (e.g., FYY becoming inaccurate starting around K=15) depends in part on the heuristic method for finding the inflection point. For example, in the Results section it was stated that “The inflection point was chosen as the first element followed by a decrease with a magnitude greater than 25% of the average decrease for the $\hat{k}$ vector.” If this heuristic was modified somehow, e.g., use 50% instead of 25%, FYY might have become inaccurate starting at some other point (e.g., *k*_0_ = 20). Perhaps some other method for finding the inflection point such as Zhu & Ghodsi (2006) could have been used instead.

If it is known or at least assumed that the underlying components are orthogonal, then Velicer’s MAP or Minka’s Laplace-PCA method might be best to use. These two approaches use PCA, which forces components to be orthogonal. And indeed Figure 1 shows that these methods achieved high accuracy on synthetic data where the true underlying components were forced to be orthogonal. However, the results indicate that in the general case of non-orthogonal components, none of the methods for estimating *k* assessed in this study seemed to work very well.

With respect to normalization methods, the various methods for estimating the number of underlying components *k* produced widely differing estimates. The results indicate that although normalization may speed up processing [21], it has an unpredictable effect on the estimation of the number of components *k*. We therefore recommend that, at least for the purpose of estimating *k*, data not be normalized. Indeed, this is the approach that has been taken in much of the public health and environmental science literature using NMF [5], [6], [8], [9].

We suggest four possible areas for future study. First, it was noted in the second study results that for the larger values of *k*_0_, none of the methods achieved high accuracy. So, a possible future study might be to fix *k*_0_ at one of these values, fix the number of genes at 60 while allowing the number of observations (subjects) to vary, and determine whether the estimate of *k*_0_ becomes better for a larger number of observations. The experiment might be repeated, but with the number of observations fixed at 1000, and with the number of genes allowed to vary. A second possible future area of work is to examine the effectiveness of *ensembles* of methods for evaluating $\hat{k}$ in order to enhance accuracy or precision. That is, one might run multiple methods for evaluating $\hat{k}$, and from the multiple results from some sort of “consensus” selection for $\hat{k}$ by weighting combinations of the results of the multiple different methods. Third, another possible future project might be to apply one or more of the methods for estimating *k*_0_ described in this study to real microarray data (e.g., the leukemia data of Golub et al., 1999 [46]), characterize the data using NMF and selecting a value for $\hat{k}$, and then use those results to simulate microarray data with $\hat{k}$ components. Finally, this study was designed to evaluate the accuracy of algorithms for identifying the number of components, and we have started with the simpler case of orthogonal components. However, because we simulated orthogonal components, we naturally gave an advantage to methods that work on orthogonal components. However, it is likely that real-world data present more complex cases where components are not orthogonal. Thus, future simulation studies should be carried out on cases where components are non-orthogonal.

One limitation of this study is that we focus on older NMF methods which have more stable and efficient implements, which have been more extensively used, and with which practitioners are more familiar. However, we recognize that the last two decades have seen an explosion of techniques based on NMF. Indeed, these developments include extensions of NMF that include sparseness constraints so that overcomplete data can be modeled [51], new divergence measures [52]–[54], and multiple algorithms to address signal-dependent noise [55]. Others have examined NMF extensions on the basis of sparseness and other constraints for graphical analysis [56] and deeply enhanced weighted NMF [57]. Even more recent work has leveraged NMF in the context of deep learning [58]–[60]. These newer techniques have not been used as extensively and have not been included here. Nevertheless, future simulation studies could include these newer methods, especially to address questions related to data normalization. Finally, another limitation of this study is that, although we focus on a handful of metrics for estimating *k*, we do not include information-based criteria like AIC or BIC (e.g. [61]). Future work could evaluate the accuracy of selecting *k* on the basis of these additional criteria.

In conclusion, for the purpose of estimating *k*_0_, we recommend that no normalization be performed. If one is willing to assume that the underlying components are orthogonal, then it may be reasonable to use Velicer’s MAP or Minka’s Laplace-PCA method. Otherwise, we recommend using methods for estimating the number of underlying components with great caution. Perhaps several methods should be tried for any given data set.

## Figures and Tables

**Table 1.** This table shows the estimates of *k* for eight normalization methods (columns) using ten methods (rows).

*k* Estimation Method	Normalization method							
	None	Scale Cols then norm rows	Subtract Mean By Rows then Std to 1	Subtract Mean By Columns then Std to 1	Subtract Global Mean then Std to 1	Subtract means by rows	Subtract mean by columns	Subtract mean by rows then by columns
Velicer	20	9	10	20	20	15	20	15
Minka-Laplace	27	17	15	25	27	27	27	27
Minka-BIC	70	70	70	70	70	70	70	70
FYV	4	4	4	4	4	4	4	4
BIC1	4	4	4	4	4	4	10	4
BIC2	4	4	4	4	4	4	10	4
BIC3	4	4	4	4	4	4	10	4
RRSSQ	4	8	4	4	4	4	10	12
BCV	18	10	24	20	14	16	12	16
CCC	18	10	24	20	14	16	12	16

## Data Availability

The Golub et al. (1999) dataset leukemia genetic data can be accessed on GitHub at this address: https://github.com/ramhiser/datamicroarray/wiki/Golub-(1999)

## Appendix: Generation of Synthetic Data

Below is MATLAB code implementing the hybrid method for generating synthetic data. Kim and Park’s basic method was combined with Cichocki et al.’s idea of using orthogonal components. To induce correlations in the synthetic data, the Cholesky decomposition of the desired correlation matrix is provided as an input matrix **U**. The definition of the function StepFunctionColumns is shown on the next page.

function [ simData ] = CorrelatedSyntheticData1(m,n,k,sparsity,noiseMag,U)
% m : Number of rows.
% n : Number of columns.
% k : Number of components.
% sparsity : Sparsity level (e.g., 0.4 for 40% sparsity)
% noiseMag : magnitude of the noise (e.g., 0.05 for 5% of the average magnitude
% of elements in simulated data
% U : Cholesky decomposition of the Correlation Matrix C; U = chol(C);
% we randomly constructed k× matrix H with 40% sparsity. (Park and Kim)
H = rand(k,n); % Uniformly distributed in [0,1]
keepH = rand(k,n); % Uniformly distributed in [0,1]
H(keepH<=sparsity) = 0.0;
% Set W to a series of orthogonal step functions.
% (Following Cichocki et al.’s “signal processing” approach.)
W = StepFunctionColumns(m,k);
% Apply the correlations to the data to the W matrix.
W = W * U;
% Compute A (Park and Kim, and also Cichocki et al., construct A in this way).
A = W * H;
% and added Gaussian noise to each element where the standard
% deviation is 5% of the average magnitude of elements in A. (Park and Kim)
avgMagA = mean(A(:)); % Compute average of elements of A.
mag = noiseMag * avgMagA; % 5% of the average magnitude of elements in A
A = A + (mag * randn(m,n)); % add Gaussian noise
simData = abs(A); % Make sure that A is non-negative after adding Gaussian noise.
function W = StepFunctionColumns(nRows, nCols)
%
% This function generates a matrix whose rows are simple orthogonal step functions
%
% INPUTS:
% nRows - desired number of rows
% nCols - desired number of columns
% 
% OUTPUT:
%
% W - matrix whose rows are simple orthogonal step functions
% If nRows < nCols, exit out with error.
if (nRows < nCols)
error(‘StepFunctionRows: nRows must be greater than or equal to nCols!’)
end
W = zeros(nRows,nCols);
% Loop over columns, design step functions
stepFactor = nRows / nCols;
startIndexTmp = 1; % Initialize startIndex.
for i=1:nCols
   % Compute endIndex from startIndex.
   endIndexTmp = startIndexTmp + stepFactor − 1;
   startIndex = round(startIndexTmp);
   endIndex = round(endIndexTmp);
   % If this is the last column, make sure the 1’s should go all the way to the last row.
   if (i == nCols)
   endIndex = nRows;
   end
   % Set select elements in the i-th column of W to 1.
   W(startIndex:endIndex,i) = ones(endIndex-startIndex+1,1);
   % Update startIndex.
   startIndexTmp = endIndexTmp + 1;
end
